# Sequence analysis of two alleles reveals that intra-and intergenic recombination played a role in the evolution of the radish fertility restorer (Rfo)

**DOI:** 10.1186/1471-2229-10-35

**Published:** 2010-02-24

**Authors:** José R Hernandez Mora, Eric Rivals, Hakim Mireau, Françoise Budar

**Affiliations:** 1 Institut Jean-Pierre Bourgin, UMR1318 INRA-AgroParisTech, Bâtiment 7, INRA Centre de Versailles-Grignon, Route de St-Cyr (RD10), 78026 Versailles Cedex France; 2 Laboratoire d'Informatique de Robotique et de Microélectronique, CNRS/Université Montpellier II, 161 rue Ada, 34392 Montpellier, France

## Abstract

**Background:**

Land plant genomes contain multiple members of a eukaryote-specific gene family encoding proteins with pentatricopeptide repeat (PPR) motifs. Some PPR proteins were shown to participate in post-transcriptional events involved in organellar gene expression, and this type of function is now thought to be their main biological role. Among PPR genes, restorers of fertility (Rf) of cytoplasmic male sterility systems constitute a peculiar subgroup that is thought to evolve in response to the presence of mitochondrial sterility-inducing genes. Rf genes encoding PPR proteins are associated with very close relatives on complex loci.

**Results:**

We sequenced a non-restoring allele (L7rfo) of the *Rfo *radish locus whose restoring allele (D81Rfo) was previously described, and compared the two alleles and their PPR genes. We identified a ca 13 kb long fragment, likely originating from another part of the radish genome, inserted into the L7rfo sequence. The L7rfo allele carries two genes (*PPR-1 *and *PPR-2*) closely related to the three previously described PPR genes of the restorer D81Rfo allele (*PPR-A*, *PPR-B*, and *PPR-C*). Our results indicate that alleles of the *Rfo *locus have experienced complex evolutionary events, including recombination and insertion of extra-locus sequences, since they diverged. Our analyses strongly suggest that present coding sequences of *Rfo *PPR genes result from intragenic recombination. We found that the 10 C-terminal PPR repeats in *Rfo *PPR gene encoded proteins result from the tandem duplication of a 5 PPR repeat block.

**Conclusions:**

The *Rfo *locus appears to experience more complex evolution than its flanking sequences. The *Rfo *locus and PPR genes therein are likely to evolve as a result of intergenic and intragenic recombination. It is therefore not possible to determine which genes on the two alleles are direct orthologs. Our observations recall some previously reported data on pathogen resistance complex loci.

## Background

The analysis of the *Arabidopsis thaliana *genome sequence led to the discovery of the Pentatricopeptide Repeat (PPR) protein family, which has undergone a spectacular expansion in land plants [1-3]. PPR proteins are composed of tandem repeats of degenerate 35 amino acid motifs. These reiterations are thought to constitute protein-RNA interaction surfaces [3,4]. Most PPR proteins are predicted to be transported to mitochondria and/or plastids [3], where they participate in various mRNA maturation steps (reviewed in [5-7]). The PPR protein family has been classified into two subfamilies. The PPR-P subfamily contains proteins uniquely formed of canonical (35 amino acid) PPR repeats, and its members were identified in plants and non-plant eukaryotes. PPR-P proteins were shown to be involved in various steps of mRNA expression like translation [8-10], intron splicing [11-14], mRNA stabilization [9,15], and RNA cleavage [13,16,17]. Proteins belonging to the PPR-PLS subfamily are specific to land plants and carry, in a defined order, repeats of slightly different sizes (called L or S) in addition to the originally identified 35 amino acid P motif. Most PLS proteins have conserved extensions at their C-terminal, such as E+ or DYW domains which were linked to RNA editing and cleavage [3,16,18-24]. Rivals et al proposed that evolution by internal duplication of blocks of PPR motifs explains the structure of PPR proteins belonging to the plant combinatorial and modular (PCMP) sub-family [25].

Recently, a comparison between the complete set of PPR proteins from three plant species indicated that almost every Arabidopsis PPR gene has a single putative ortholog in *Oryza sativa *(rice), showing that PPR proteins have a high degree of interspecies conservation between monocots and dicots. The sequences of two groups of PPR-P proteins could not be aligned between Arabidopsis and rice and these genes represent distant homologues of fertility restorers of cytoplasmic male sterility identified in radish and rice [4]. *Restorers of fertility *(or *Rf*) are nuclear genes that prevent the action of non-conserved and often chimeric mitochondrial genes that cause cytoplasmic male sterility (CMS). CMS sterility-inducing genes and their corresponding *Rf *are the genetic factors of the best theoretically analyzed genomic conflict in plants [26]. CMS systems have also been widely used in the production of hybrid crops [27] and as a model for studying nucleo-mitochondrial interactions [28]. Since the identification of the first *Rf *gene in Petunia [29], *Rf *genes encoding PPR-P proteins were identified in rice [30-32] and radish [33-35]. Interestingly, *Rf *genes are carried on complex loci, containing several closely related genes, generally unable to restore fertility. For example, the restoring allele of the radish *Rfo *locus, here named D81Rfo, carries three related PPR genes arbitrarily named *PPR-A*, *PPR-B*, and *PPR-C *[33-35]. The *PPR-B *gene confers the fertility restoration activity, whereas *PPR-A *and *PPR-C *do not [10,33-35]. *PPR-C *was shown to be a pseudogene [10]. Several related PPR genes are also clustered on the rice genome at the *Rf-1 *locus [31,36,37]. This led to the idea that *Rf *genes, unlike other PPR genes, might undergo an evolutionary process recalling that of resistance genes in plants [38]. Resistance genes are arranged in complex clusters and are thought to evolve through a birth-and-death mechanism [39,40].

By analyzing the rice *Rf-1 *locus in a large number of *Oryza *lines from wild and cultivated species, Kato et al showed that the ancestral *Rf-1 *gene likely underwent duplication in an ancient progenitor of the *Oryza *species AA genome and that then intergenic homologous recombination probably contributed to the diversification of alleles [36].

Geddy and Brown analyzed syntenic genomic regions from *Arabidopsis thaliana *and *Brassica rapa *or radish and showed that the location and direction of PPR genes are less conserved than those of non-PPR genes of the same regions, and therefore qualified them as "nomadic" [41]. They also suggested that interallelic recombination could be the mechanism leading to the observed variability in copy number and sequence among PPR genes.

In this report, we describe the sequence and genetic organization of a non-restoring allele of the *Rfo *locus (L7rfo), isolated from a European radish cultivar that was selected for the absence of restorers [42]. By comparing it with the previously described restorer allele (D81Rfo), originating from an Asian genotype, some interesting observations could be made which strongly suggest that several mechanisms acted in the diversification of *Rfo *alleles. These include recombination and insertion of sequences originating from other locations in the radish genome. We describe two new PPR genes that are closely related to *PPR-A*, *PPR-B*, and *PPR-C *and investigate their phylogenetic relationship. Our results reveal that these five related PPR genes share a common sequence organization, probably present in their common ancestor. We report evidence that some of these genes originate from intragenic recombination. We also identify an internal duplication of a sequence fragment encoding 5 PPR repeats in the 3' end of the genes.

## Results

### Cloning and sequencing of a non-restoring allele of the *Rfo *locus from a European radish

In order to obtain the complete sequence of a non-restorer (also named maintainer) allele (*L7rfo*) for the *Rfo *locus, we constructed a BAC library from the L7 radish line, which was selected from European radish cultivars for maintaining Ogura sterility [42]. Genomic clones carrying the *L7rfo *allele were selected using two PCR markers closely linked to the *Rfo *locus and previously identified during identification of the restorer allele (*D81Rfo*)[34]. These markers amplify parts of genes flanking the PPR genes of the *Rfo *locus (see Methods section for details). A single clone containing both markers was selected and completely sequenced. The sequence of the corresponding 41,492 bp DNA insert was deposited in Genbank (accession number FN397617). Thereafter, this sequence will be named L7rfo, whereas the previously described sequence of the restorer genotype derived from an Asian cultivar will be designated D81Rfo (accession number AJ550021). *Rfo *will be used to designate the locus or to make general statements applying to both alleles.

### The L7rfo locus carries two PPR genes and is not entirely collinear with Rfo

The L7rfo and D81Rfo sequences were compared by local pairwise alignment using the YASS program [43]. The sequence available for D81Rfo is longer than the L7rfo sequence and extends on both sides of it, thus we only analyzed the DNA regions for which sequence information was available for both genotypes. Genes were predicted using GENSCAN [44]. The *Arabidopsis thaliana *protein database was screened with the peptide sequences of the predicted gene products using the BLASTP program [45] and predicted genes that do not have a homolog in Arabidopsis were disregarded.

**Figure 1:**
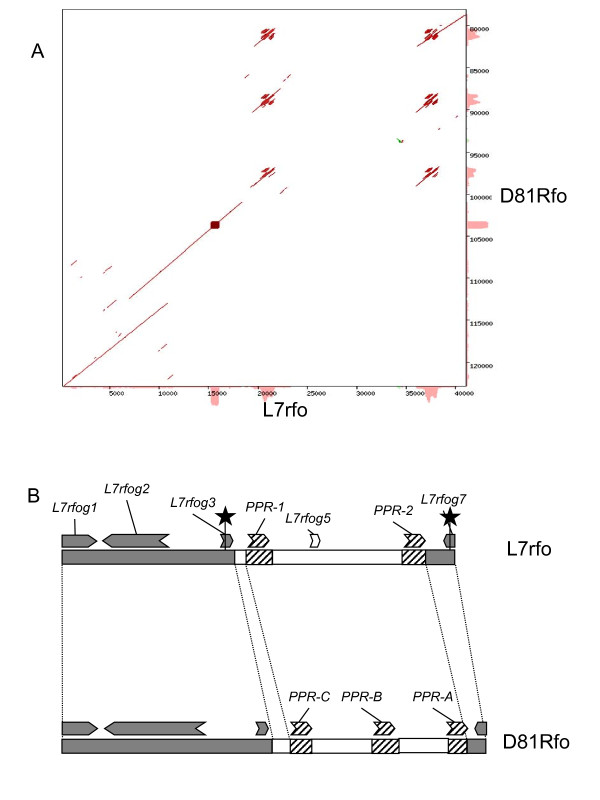
**Sequence comparison of both alleles of the *Rfo *locus**. L7rfo is the sequence determined in this study (accession number FN397617) and D81Rfo is the corresponding region from the BAC64 clone (accession number AJ550021) [34]. A- Dot Matrix view of the YASS comparison of the two sequences using default parameters except that the E-value threshold was 10^-10^. B- Schematic representation of both alleles. Black stars indicate the positions targeted by PCR markers used to screen the BAC library. The dark grey boxes highlight collinear and highly similar regions. The hatched boxes indicate regions that are homologous but not collinear. The white boxes indicate the L7rfo region that has no corresponding sequence on D81Rfo, and vice-versa. Arrows symbolize genes predicted by GENESCAN and indicate the direction of transcription. Unlabelled predicted genes on D81Rfo are highly similar to the corresponding genes on L7rfo, and were previously reported by [34]. Gene information is provided in Table 1.

The results are summarized in Figure 1 and Table 1. Comparison of both sequences shows that three classes of regions can be distinguished. Firstly, two highly similar regions (92% to 97% identity) of 18,350 bp and 22,166 bp, and 3,164 bp and 1,976 bp in L7rfo and D81Rfo respectively, were detected by YASS with an E-value threshold of 10^-10^. They are collinear except for one duplication that is present in the disease resistance gene carried by the D81Rfo sequence but not in that carried by L7rfo. These two regions include the PCR markers used to screen the BAC library and homologues of the genes from the corresponding syntenic region of Arabidopsis chromosome 1 (Table 1). Secondly, two regions of L7rfo showed 90 to 94% identity with the region of D81Rfo carrying the *PPR *genes and two *PPR *genes, named *PPR-1 *and *PPR-2*, were predicted. Lastly, a large central region in L7rfo (13,722 bp) and a small region upstream of *PPR-1 *(1,163 bp) showed no clear similarity with the D81Rfo sequence. In addition, a small region in D81Rfo (1,936 bp) upstream of *PPR-C*, and two regions located between adjacent *PPR *genes share no homology with the L7rfo sequence. Pairwise comparisons using different E-value thresholds for YASS or the BLASTN program gave similar results, except that the lengths of the different types of regions were slightly different.

**Table 1:** Predicted genes in the L7rfo sequence. See also Figure 1

Name on Figure 1	Start/stop positions	Homologue on Rfo sequence	Closest Arabidopsis homologue	Function of protein encoded by Arabidopsis homologue
L7rfog1	<1-3656	yes	At1 g63770	Putative amino-peptidase
L7rfog2	11320-4352	yes^a^	At1 g63740	Disease resistance
L7rfog3	16809-18107	yes	At1 g63720	unknown
PPR-1	19640-21812^b^	yes	At1 g64100	unknown
L7rfog5	26247-27279	no	At1 g35320	Innate immunity
PPR-2	36144-38325^b^	yes	At1 g64100	unknown
L7rfog7	>41492-40463	yes	At1 g63680	UDP-N-acetylmuramoylalanyl-D-glutamate-2,6-diaminopimelate ligase

^a^The closest Arabidopsis homologue of the corresponding gene present on the Rfo sequence is At1g63730.

^b^*PPR-1 *and *PPR-2 *predictions from GENESCAN were corrected after pairwise alignments with PPR-A, PPR-B and PPR-C.

**Figure 2:**
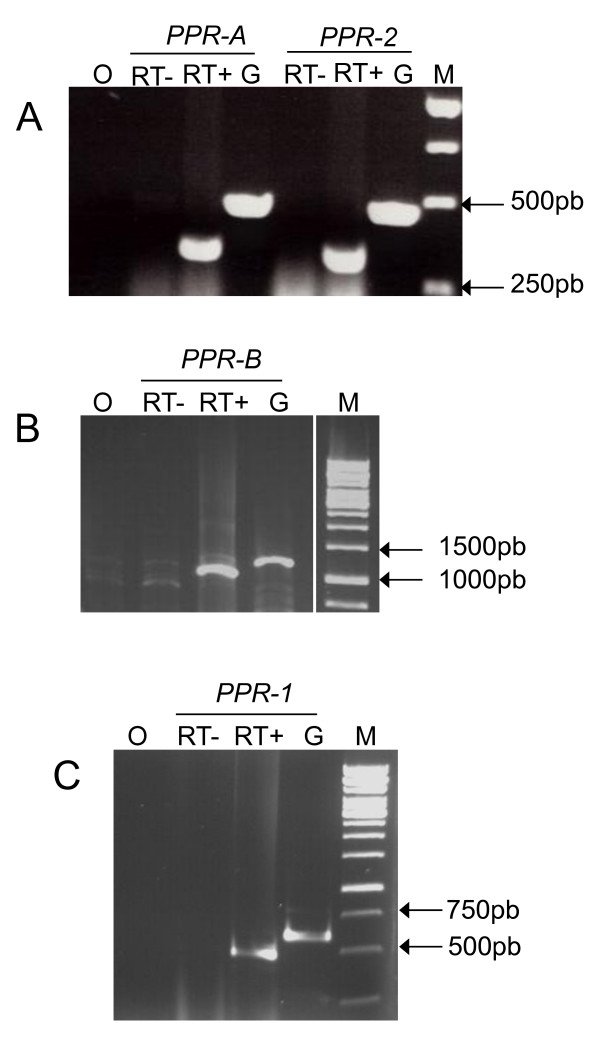
**Expression analysis of genes encoding PPR proteins on both alleles of the Rfo locus**. RT-PCR were carried out on total RNA from radish flower buds using primers specific for each gene and electrophoresed on a 1% agarose gel. Lanes O: negative PCR control (no substrate); lanes RT-: control without reverse transcriptase on DNAse treated RNA before amplification; lanes RT+: RT-PCR reaction; lanes G: PCR amplification from genomic DNA; lanes M: molecular size standards (GeneRuler 1 kb DNA ladder, Fermentas). A. The primers used were PPRA:20505U22 and PPRA:20954L21, which amplify *PPR-A *in D81Rfo and *PPR-2 *in L7rfo. B. The primers used were Rfocons1047U22 and PPRB:13225L22, which amplify *PPR-B *in D81Rfo. C. The primers used were PPR1:21229U22 and PPR1:21229U22, which amplify *PPR-1 *L7rfo.

RT-PCR analyses indicated that both *PPR-1 *and *PPR-2 *are transcribed, at least in flower buds, and sequencing of amplification products confirmed the presence of an intron in their 3' regions, as in *PPR-B *and *PPR-A *(Figure 2, additional File 1). Like the D81Rfo PPR genes, *PPR-1 *and *PPR-2 *are predicted to encode proteins containing 17 PPR-P repeats. Interestingly, the putative PPR-1 and PPR-2 proteins contain the same four amino acid deletion in the third PPR repeat also found in PPR-A [34]. PredOtar [46] and TargetP [47] subcellular targeting prediction programs both predicted that the putative PPR-1 and PPR-2 proteins are transported to mitochondria (data not shown).

### Analysis of phylogenetic relationships between PPR genes of the *Rfo *locus

**Figure 3:**
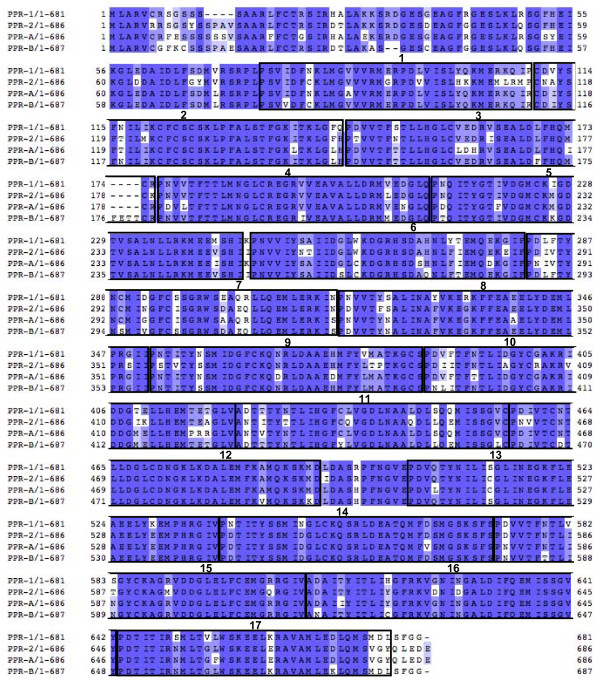
**Global alignment of PPR-1, PPR-2, PPR-A, and PPR-B protein sequences**. The alignment was performed with MUSCLE (v3.7) with default settings on the Phylogeny.fr platform and edited with JALVIEW [69]. The residues are colored according to percentage identity, from dark blue: 100% identity to white: less than 50% identity. PPR motifs predicted by the cyclic hidden Markov model program at http://atgc.lirmm.fr/PPR/[59] are framed and numbered.

**Figure 4:**
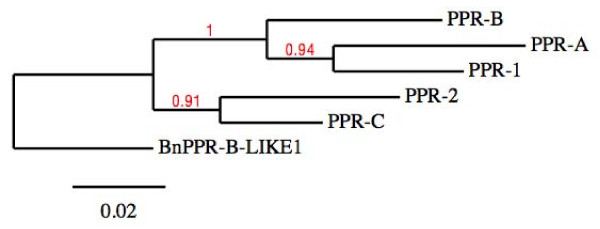
**Phylogenetic tree of genes from the *Rfo *alleles**. The sequence of the related gene *BnPPR-B-LIKE1 *from *Brassica napus *(accession number FJ455099) [10] was used as an outgroup to root the tree. The analysis was performed on the Phylogeny.fr platform (see details in Material and Methods). Reliability for internal branches was assessed using the bootstrapping method (500 bootstrap replicates). Bootstrap results are indicated in red. Graphical representation and editing of the tree were performed with TreeDyn (v198.3) [70].

We compared the sequences of the putative PPR-1 and PPR-2 proteins with PPR-A and PPR-B; *PPR-C *was not included in this analysis because it is a pseudogene [10]. The alignment indicates that all four proteins are closely related and likely arose from a common ancestor, although only PPR-B possesses a complete third PPR motif (Figure 3). The percentage of identity between the PPR protein sequences encoded by the *Rfo *locus, is above 84%, which precludes phylogenetic analyses on peptide sequences. Therefore, in order to infer the phylogenetic relationship of the PPR genes present at the *Rfo *locus, we carried out multiple global alignments of their coding sequences. This meant that the *PPR-C *pseudogene could also be included (Additional File 1). A maximum likelihood phylogenetic tree was constructed with PHYML from the MUSCLE alignment obtained with the five sequences plus the sequence of the closely related rapeseed gene *PPRB-LIKE1 *as the outgroup [10] (accession number FJ455099) (Figure 4).

The resulting tree showed that, although the *PPR-1 *and *PPR-2 *genes are from a European radish cultivar, they group with *PPR-A *and *PPR-C*, respectively, which were sequenced from an Asian radish genotype. This result suggests that a PPR gene duplication at the *Rfo *locus predates the divergence of Asian and European genotypes, and that *PPR-1 *and *PPR-A*, on one hand, and *PPR-2 *and *PPR-C*, on the other hand, probably derived from two distinct copies of the *Rfo *PPR gene in the common ancestor of these two geographically isolated radish genotypes.

### Intragenic recombination appears to have occurred during the evolution of *Rfo *PPR genes

**Figure 5:**
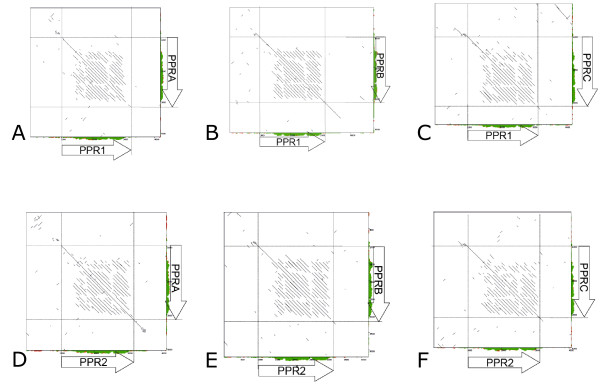
**Comparison of flanking regions of the PPR genes from the *Rfo *locus**. Pairwise comparisons were made between the two genes of the L7rfo sequence and the three genes of the D81Rfo sequence using YASS with default settings. The genes (from initiation to stop codons) are represented as arrows along the sequence scales. Vertical and horizontal lines indicate gene borders on the dot plots. A: *PPR-1 *vs *PPR-A*; B: *PPR-1 *vs *PPR-B*; C: *PPR-1 *vs *PPR-C*; D: *PPR-2 *vs *PPR-A*; E: *PPR-2 *vs *PPR-B*; F: *PPR-2 *vs *PPR-C*.

The respective positions of the L7rfo and D81Rfo PPR genes on either allele are not in good agreement with the phylogenetic analysis (see Figure 1 and Figure 4). This observation suggests that sequence rearrangements occurred during allele differentiation at the *Rfo *locus. This led us to carry out a pairwise alignment of the L7rfo PPR genes, including 1 kb of flanking sequence, versus the three D81Rfo genes and their flanking sequences (Figure 5). The results showed that short fragments of ca 150 bp of the upstream regions flanking *PPR-1*, *PPR-2*, *PPR-B *and *PPR-C *are similar, whereas no similarities were found between downstream sequences, except between *PPR-1 *and *PPR-B*, and *PPR-2 *and *PPR-A*. Interestingly, the multiple gene alignment clearly showed that the 3' sections of the *PPR-1 *and *PPR-B *genes, including the introns, are shared. A similar observation was also made for *PPR-2 *and *PPR-A *(Additional File 1).

**Figure 6:**
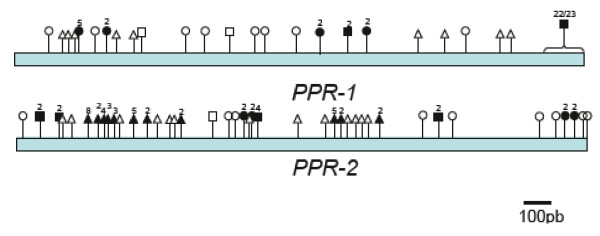
**Schematic representation of shared nucleotide polymorphisms along the PPR-1 and PPR-2 coding sequences**. The multiple gene alignment (additional file 1) was used to detect shared polymorphisms between *PPR-1 *or *PPR-2 *and the genes from the D81Rfo sequence. Flags represent positions of polymorphisms shared by the considered gene and *PPR-A *(circles), *PPR-B *(squares), or *PPR-C *(triangles). Open forms indicate a single position. Filled forms indicate several positions that were too close on the sequence to be distinctly represented on the diagram. The numbers above filled forms indicate the number of positions concerned. The end section of *PPR-1*, which contains the intron sequence, is treated as a block sharing 22 polymorphisms with *PPR-B *and 1 with *PPR-C *(regions with gaps were not considered).

We therefore analyzed the similarities within the gene coding regions of *PPR-1 *and *PPR-2 *and their relatives on the D81Rfo allele in further detail. A multiple alignment of gene sequences (Additional File 1) revealed polymorphisms shared between *PPR-1 *or *PPR-2 *and the genes from the D81Rfo sequence (Figure 4). We considered that a polymorphism was shared between two sequences when the nucleotide at this position was identical between the two considered sequences and different from that present at the same position in the three other genes (regions with gaps were not considered). For each gene, we observed that polymorphisms shared with a gene of the D81Rfo allele were grouped together rather than being spread along the sequence. It was particularly obvious for polymorphisms shared between the ends of *PPR-1 *and *PPR-B*, the first halves of *PPR-2 *and *PPR-C*, and the ends of *PPR-2 *and *PPR-A *(Figure 6). This analysis suggests that different parts of *PPR-1 *and *PPR-2 *share a most recent common ancestor with different genes of the D81Rfo allele, which implies that intragenic recombination occurred during gene evolution.

**Figure 7:**
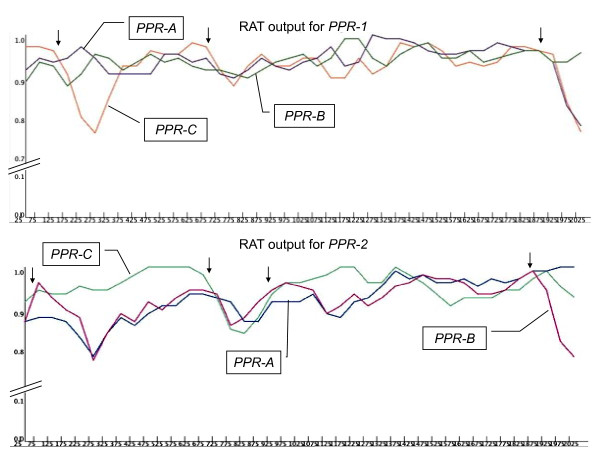
**Graphical results of the RAT program along coding sequences of *PPR-1 *and *PPR-2***. PPR-1 and PPR-2 coding sequences were analyzed according to their level of identity with *PPR-A, PPR-B*, or *PPR-C*. Vertical arrows indicate the positions of putative recombination points detected by RAT.

We identified possible recombination points in the two genes by using the RAT program [48], which was designed to infer recombination points by detecting abrupt changes in the similarity profile of a target sequence (Figure 7). Analysis of the *PPR-1 *coding sequence revealed putative recombination points between positions 125 and 175, positions 675 and 725, with *PPR-A *as the closest relative between these two points, and positions 1875 and 1925. Analysis of the *PPR-2 *coding sequence revealed putative recombination points in the following regions: [25,75], [675, 725], with PPR-C as the closest relative between this two points, [875, 925] and [1875, 1925]. These results support our conclusion that intragenic recombination appears to have occurred among PPR genes of the *Rfo *locus during evolution.

### The sequence of PPR genes from the Rfo locus results from internal duplication of PPR repeat coding regions

**Figure 8:**
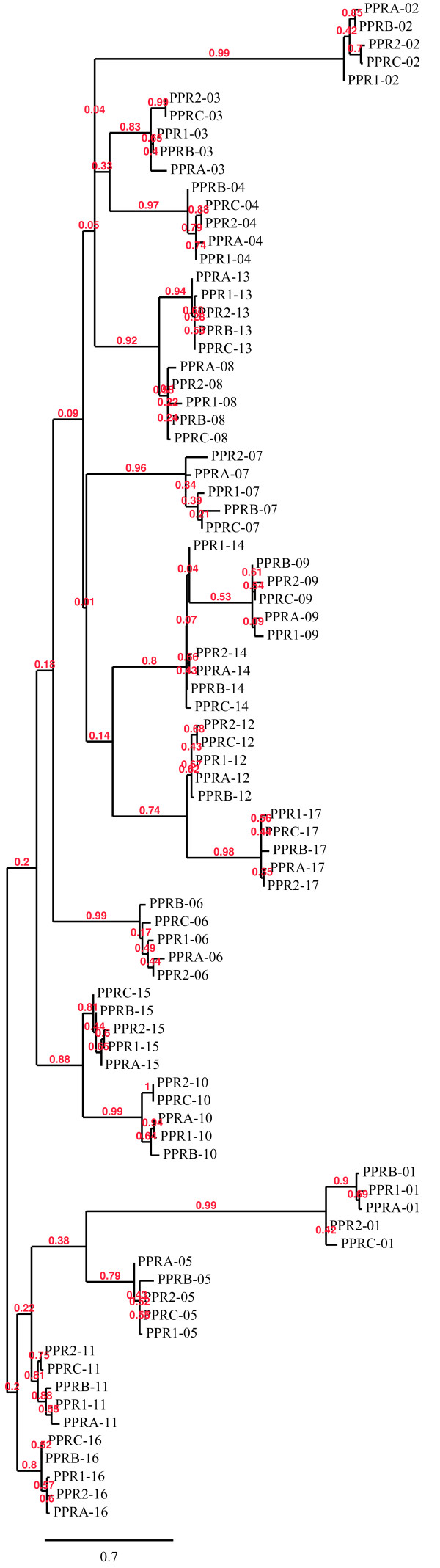
**Maximum likelihood phylogenetic tree resulting from multiple alignment of PPR repeat coding sequences of the 5 *Rfo-PPR *genes**. The tree was constructed with TreeDyn (v198.3) [70] on the Phylogeny.fr platform after multiple alignments were conducted as described in the Material and Methods section. Bootstrap values obtained after 100 repetitions are shown.

In a recent report, the PCMP (PLS) sub-family of PPR genes was proposed to have arisen via duplication of PPR motif coding regions [25]. We took advantage of the availability of sequences of five highly related genes to test whether information regarding the structure of their common ancestor could be obtained by comparing their PPR repeat coding regions. We carried out multiple alignments with the PPR motif coding sequences from the 5 PPR genes from L7rfo and D81Rfo using MUSCLE. Each sequence used was identified according to its gene of origin (*PPR-1*, *PPR-2*, *PPR-A*, *PPR-B*, *PPR-C*) and the position of the repeat in the protein (01 to 17). For this purpose, only coding sequences were considered and the intron in repeat 17 was removed. A phylogenetic tree was constructed with PHYML using the GTR model (Figure 8). Each PPR motif coding sequence was found to be associated with coding sequences of the corresponding repeats in the 4 other genes. This shows that the structure is conserved among the 5 genes. It suggests that the general motif structure of the common ancestor of the five genes was most probably the same. In addition, the coding sequences of repeats 8 and 13, 9 and 14, 10 and 15, 11 and 16, and 12 and 17 appear more related to each other than with any other PPR motif coding sequence. Furthermore, their consecutive positions in the genes strongly suggest that the sequence fragments encoding repeats 8, 9, 10, 11, and 12, and that encoding repeats 13, 14, 15, 16 and 17, result from a tandem duplication event.

It should be noted that when the same analysis was carried out without removing the intron from the coding sequences of repeat 17, the results did not change significantly (data not shown). Finally, when we included repeat coding sequences from the *B. napus *related gene *PPR-B-LIKE1*, we observed that each repeat of the rapeseed gene clustered with the corresponding repeats of the radish genes, indicating that the gene structure was conserved throughout speciation (Additional File 2).

## Discussion

Here we describe the *Rfo *region from a non-restoring (maintainer) genotype of radish (L7rfo) that was analyzed by comparison with the previously described counterpart region from a restorer genotype (D81Rfo) [33,34]. At least one *Rfo*-related gene has been reported outside the *Rfo *locus in the restorer cultivar, (g1 in [41]), thus it was important not to rely on PPR-gene sequences to isolate the L7rfo allele. On the contrary, we used PCR markers anchored in radish homologues to At1 g63720 and At1 g63680 to isolate the RfoL7 allele, as did Desloire et al to isolate the D81 allele [34]. Homologs to At1g63770, At1 g63720, At1 g63730, and At1 g63680 were identified on the L7rfo sequence, as on D81Rfo. In addition, markers derived from At1 g63770, At1g63720, At1 g63730, and At1g63680 gene sequences, for which homologs were identified in L7rfo and D81Rfo sequences, were used during the fine mapping of the *Rfo *locus in the cross D81xL7 [34]. Therefore the allelism of D81Rfo and L7rfo is not doubtful. The two currently available allele sequences originated from an Asian (for D81Rfo) and a European (for L7rfo) genotype. We previously reported that the Ogura CMS probably followed different evolutionary pathways in Asian and European lineages [49]. The two alleles compared here might therefore diverge since quite a long time, but, in any case, they are expected to be more closely related than loci from related species, as the *Rf-1 *loci studied in different rice genomes by Kato et al [36].

The first obvious finding from the D81Rfo vs L7rfo comparison is that two PPR genes, separated by an unrelated gene, are found on L7rfo, whereas D81Rfo carries three PPR genes in tandem. Secondly, a global alignment showed a break in colinearity between the two sequences (Figure 1). The robustness of this observation to changes in the E-value threshold in the Yass program led us to define the boundaries of the complex *Rfo *locus as the colinearity break points, although the exact position of these points obviously depends on the alignment stringency. In addition, we detected sequences on each allele with no homologous counterpart on the other (white boxes in Figure 1). Although the lengths of such "unique" sequences upstream *PPR-1 *and *PPR-C *may be reduced with a less stringent E-value threshold, there is no doubt that a large region between *PPR-1 *and *PPR-2 *in L7rfo is absent in D81Rfo. Furthermore, the predicted gene in this region (*L7rfog5*) is homologous to the Arabidopsis *MOS2 *gene, which is involved in plant innate immunity [50]. The Arabidopsis genome contains two similar *MOS2 *genes, At1 g33520 and At4 g25020, the former being the closest homologue to *L7rfog5*, but none is located in the Arabidopsis genomic region syntenic to the *Rfo *locus [34] (Table 1). Therefore, this central region in the L7rfo allele is unlikely to originate from a location close to the *Rfo *complex locus and probably results from an insertion in the L7rfo allele rather than a deletion in D81Rfo. This insertion could have resulted from illegitimate recombination occurring in intergenic regions between non allelic sequences, thus we searched for repeated sequences that could be involved in this type of mechanism. Among others, we found a short (35 nt) direct repeat sequence immediately upstream of the *PPR-2 *gene, and an imperfect indirect repeat sequence approximately 600 bp upstream of the *L7rfog5 *gene (data not shown). Nevertheless, it is difficult to determine if and how these repeats were involved in the mechanism. To date, no such insertions inside a complex *Rf *locus were reported, however the previous comparisons of complex *Rf *loci would probably not have detected such insertions. In particular, the comparison of rice *Rf-1 *alleles was conducted by PCR analysis [36], and it is possible that in some cases the *Rf-1 *allele could not be entirely defined by PCR amplification because insertions from other regions of the genome could have separated the different PCR primer binding sites. Analysis of complete allele sequences of other *Rf *complex loci will determine whether foreign sequences have also inserted in *Rf*-PPR clusters from other species.

We focused our sequence analyses on the two PPR genes carried by the L7rfo allele. The gene predictions obtained from GENSCAN were not very accurate, so we aligned the coding sequences of *PPR-1 *and *PPR-2 *with those of the closely related genes from the D81Rfo sequence. We detected RNAs corresponding to these genes by RT-PCR (Figure 2), indicating they are expressed, at least at the RNA level. An antibody raised against the PPR-B protein did recognize proteins of the expected size in extracts from the L7 genotype [10]. Thus, at least some of these detected proteins could be products of the *PPR-1 *and *PPR-2 *genes, although some could also be proteins encoded by related genes located outside the L7rfo allele. By sequencing RT-PCR products we determined the precise position and length of the intron in the *PPR-1 *and *PPR-2 *genes, and also confirmed those of *PPR-A *and *PPR-B *(additional File 1). The predicted protein sequences of the four genes are remarkably similar, sharing 84.4% to 89% identity (Figure 3). The analyzis of the phylogenetic relationships between the PPR genes of the *Rfo *locus (Figure.4) indicated that the genes of each allele do not derive from recent duplications independently in the European and Asian lineages. Therefore, a duplication of an *Rfo*-PPR gene probably occurred in the common ancestor of the Asian and European radish genotypes. The genes found on different alleles of the rice *Rf-1 *locus were also proposed to derive from ancient duplications [36]. However, there are striking differences between the findings reported regarding the comparison of rice *Rf-1 *regions and our comparison of radish *Rfo *alleles. Kato et al. [36] reported that flanking sequences specific to each *Rf-1 *gene as well as the gene order between clusters from different species were conserved. Here we observed almost no conservation of flanking sequences between the *Rfo*-PPR genes, although we compared two genotypes of the same species (Figure 5). In addition, the order of the genes relatively to the locus flanking sequences is not consistent with their phylogenetic relationships inferred from the multiple alignment (Figure 1 &4). It cannot be ruled out that the PCR-based approach used to analyze diversity among the rice *Rf-1 *restricted observations to situations where the gene order was conserved. Nevertheless, the extremely reduced conservation of PPR-gene flanking sequences between the radish genotypes compared with the long stretches of conserved PPR-gene flanking sequences between rice species strongly suggests that the evolution of the radish *Rfo *locus was dramatically more dynamic and complex than that of the rice *Rf-1 *locus. As a result, it is not possible to assign orthologs for *PPR-1 *and *PPR-2 *on D81Rfo. Variable numbers and complex phylogenetic relationships between resistance genes were also described between alleles of the *Mi-1 *resistance gene clusters in tomato [51] and of the *RPP5 *locus in Arabidopsis [52]. Such observations likely result from interallelic recombination events.

Interallelic recombination can also lead to intragenic sequence exchanges that may also affect the structure of the genes by modifying the number of repeated domains, as reported for some disease resistance genes [52,53]. The petunia *rf-PPR592 *gene, sequenced from a maintainer genotype, was reported to probably result from intragenic recombination between closely related *PPR *genes [29]. The distribution of polymorphisms in the coding sequences of *PPR-1 *and *PPR-2 *appears to reveal that intragenic recombination also occurred at the *Rfo *locus (Figure 6). On the other hand, the 17 PPR repeat structure shared by *Rfo-PPR *genes seems to be remarkably stable (Figure 8). By adding the coding sequences of repeats from the related rapeseed gene to the analysis, we could show that this 17 repeat structure probably predates the divergence of the two species (Additional File 2). Interestingly, the Ogura CMS system does not exist in any other species than wild and cultivated radishes (H. Yamagishi, personal communication). Therefore, the Ogura restoring function of *PPR-B *was selected in the radish lineage probably by duplication and neofunctionalization of a previously existing PPR gene, with a 17 repeat structure. It would therefore be particularly interesting to investigate what was the biological function of the *PPR-B *ancestor gene in a species were no Ogura CMS gene operated.

An intriguing question regarding the PPR-B third PPR motif also arises. Among all the PPR genes described at the *Rfo *locus, only *PPR-B *carries a complete third PPR repeat coding sequence. PCR experiments specific for the PPR-B third PPR motif were unsuccessful with all the rapeseed genomic DNA tested (our unpublished results). All the *PPR-B *related genes with an incomplete third PPR motif carry the same deletion, so it is very unlikely that this deletion occurred independently in different gene lineages. An ancestral gene carrying a complete third PPR motif might have existed at the basis of the lineage of all PPR genes related to *PPR-B*, and the deletion in the third PPR motif might have occurred early in this lineage, the rapeseed genome retaining only genes with the deletion.

An internal duplication, as those hypothesized in the evolution of some resistance genes [52,53], seems to be involved in the formation of *Rfo-PPR *genes. The results of our phylogenetic analysis strongly suggest that the 10 C-terminal repeats of these genes result from tandem duplication of a five repeat block. This raises a question concerning the intron, located in the 3' part of the coding region of the 17^th ^repeat. The absence of intron in the 12th repeat suggests that either the intron was lost after the duplication of the repeat, or that the intron was inserted at the end of the 17th repeat at a later stage, after the gene structure had been established.

*Rf-PPR *genes were proposed to have evolved through a process similar to that of disease resistance genes [38,39]. The selective pressure involved is the need for the nuclear genome to repress male sterility-inducing genes appearing in the mitochondrial genome, in order to restore their transmission to progeny via pollen. Although few *Rf-PPR *genes have been identified so far, several features of their sequences and genomic organization emerge and appear to reinforce this idea. Among these features is the complex organization of *Rf *loci, with the presence of *PPR*-genes closely related to restorers of fertility, but unable to restore fertility. The variable number of closely related PPR genes at *Rf *loci, probably as a result of interallelic recombination and unequal cross over events is also considered as a signature of the birth-and-death evolutionary process proposed for resistance genes.

The data provided by this study completes the overall view obtained from previous analyses of different *Rf *loci. These findings confirm that a variation in the number of related PPR genes present on different alleles is a shared feature of *Rf *loci. Our results also suggest that the evolution of *Rf *alleles has involved insertion of unrelated sequences, a process that has not been previously reported, and intragenic recombination, a mechanism thought to contribute to diversification of disease resistance genes [39].

## Conclusions

By thoroughly analyzing the sequences of two divergent alleles of the radish *Rfo *restorer locus, we obtained new insight into the evolutionary peculiarities of *Rf *loci and *Rf-PPR *genes. Our results suggest that alleles at the *Rfo *locus evolved through recombination, as well as insertion of "nomadic" sequences. In particular, we provide evidence that PPR genes of the *Rfo *locus experienced intragenic recombination during their evolution. Although *Rfo*-*PPR *genes are very prone to recombination, their structure, which is composed of 17 PPR motif repeats, seems to remain unchanged and probably arose in an ancestor species of rapeseed and radish by duplication of a block of 5 repeats at the C-terminal end of the protein.

## Methods

### BAC library construction and identification of the clone carrying the rfo allele

A BAC library was constructed from the radish L7 line following the method described by Peterson et al [54]. The L7 line is a European radish line selected for the absence of restorers [42]. Nuclei were extracted from young leaves after 2 days in the dark and included in agarose plugs. After partial digestion with *Hin*dIII, fragments between 40 and 100 kb were eluted from a pulse-field agarose gel and ligated into the *Hin*dIII cloning-ready pIndigoBAC-5 vector (Epicentre Biotechnologies) and transformed into ElectroMAX DH10B electro-competent *E. coli *cells (Invitrogen). The resulting library comprised ca 23,000 BAC clones. The library was amplified in 32 pools and each pool was screened with F24D7-9rad and F24D7-13rad PCR markers (Tables 2 and 3). One pool was positive for both markers. 2688 colonies from this master pool were then further screened and one clone positive with both primer pairs was selected for sequencing. The selected BAC clone was sequenced by the Centre National de Séquençage (Evry). The sequence was named L7rfo and deposited in the EMBL nucleotide sequence database under the accession number FN397617.

**Table 2:** Primers used to screen the BAC library and RT-PCR experiments

Name of primer	5'-3' sequence
F24D7-9radF	TAAGCTGAGCGAGTGGACTACC
F24D7-9radR	AGACTATAAACGCAGCCGCTAC
F24D7-13radF	CTTGATTCGGTTCGAGAGCTTA
F24D7-13radR	TCCATGGGAACTCGCTTGTGTC
PPRA:20505U22	CTTCTCTCCCAACGTAGTGACA
PPRA:20954L21	CATTCATCCTCCAACTGATAC
Rfocons1047U22	AATTATACGATGAGATGCTTCC
PPRB:13225L22	AAACAGAAGAAAATCTTTGATC
PPR1:21229U22	GATGCCACATAGAGGTATAGTC

**Table 3:** PCR amplification conditions

Primer pair (upper primer lower primer)	purpose	Annealing temperature	Extension time
F24D7-9radR F24D7-9radF	Screening of BAC library	55.5°C	1 min
F24D7-13radR F24D7-13radF	Screening of BAC library	54°C	1 min
PPRA:20505U22 PPRA:20954L21	RT-PCR on PPR-A and PPR-2	52°C	30 sec
Rfocons1047U22 PPRB:13225L22	RT-PCR on PPR-B	52°C	1 min 15 sec
PPR1:21229U22 PPRB:13225L22	RT-PCR on PPR-1	51°C	1 min

### Expression analyses

Total RNA and genomic DNA were extracted from buds of two radish genotypes, L7 and D81, carrying the maintainer (L7rfo) and restorer (D81Rfo) alleles of the locus, respectively. Total RNA was extracted using Trizol reagent (Invitrogen). Genomic DNA was extracted as previously described [55].

Total RNAs were treated with RNAse free-DNAse (Fermentas) in the supplied buffer, at 37°C for 90 min. DNAse treatment was stopped by adding 2.5 mM EDTA (final concentration) and incubating at 65°C for 10 min. DNAse treated RNAs were then extracted with phenol/chloroform/isoamyl alcohol (25/24/1) and precipitated. The pellet was dissolved in 25 μL of water with 1 μg of dT_18 _for cDNA priming. The mix was heated to 65°C and cooled on ice. cDNA synthesis mix was then prepared as recommended by Fermentas and separated into two 19 μL aliquots just before adding the reverse transcriptase. In one aliquot reverse transcriptase was omitted (RT-control), in the other 1 μL (200U) of Fermentas Revert Aid H^-^M-MuLV Reverse Transcriptase was added (RT+ sample). Both were then incubated at 42°C for 90 min, and PCR amplifications were performed directly using one μL from each tube.

PCR amplification was conducted in 25 μL reaction volumes for 35 cycles. Annealing temperatures and extension times were adapted to each primer pair used (see Tables 2 and 3).

For sequencing of RT-PCR products, two independent PRC reactions were mixed in order to dilute out any mistakes introduced by the *Taq *polymerase, and sent to Genoscreen for sequencing with the upper PCR primer. Sequences were aligned with the genomic sequence to precisely locate intron limits.

### Definition of gene sequences and PPR repeat sequences

Genes on the L7rfo sequence were predicted by GENSCAN [56,57]. PPR genes were then defined more precisely by aligning their coding sequences with those of the *PPR*-genes of the Rfo allele. Accordingly, the prediction for *PPR-2 *was corrected and extra 5' predicted exons were discarded. The peptide sequences of non-PPR predicted gene products were then compared to the *Arabidopsis thaliana *protein database using the BLASTP program [45] and predicted genes with no Arabidopsis homologue were disregarded.

PPR motifs were defined according to a cyclic hidden Markov model program [58,59].

### Sequence analyses

Subcellular location of the PPR-gene products was predicted using PredOtar v1.03 [46,60], and TargetP v1.1[47,61].

Pairwise sequence comparisons were carried out using YASS at [43,62] with default settings, except when mentioned in the text or figure legend.

Multiple sequence comparisons were carried out on the platform at [63,64]. Alignments were carried out using MUSCLE (v3.7) [65] using default settings. After alignment, ambiguous regions (i.e. containing gaps and/or poorly aligned) were removed with Gblocks (v0.91b) [66]. The phylogenetic tree was reconstructed using the maximum likelihood method implemented in the PhyML program (v3.0aLRT) [67,68].

Intragenic recombination was analyzed using the RAT program [48] using a window size of 100 nt by increments of 50 nt, and minimum and maximum cut-off scores of 87% and 96%, respectively.

## Authors' contributions

JRH constructed the BAC library, isolated the BAC clone with the L7rfo sequence and carried out sequence analyses under the supervision of ER.

ER conducted sequence analyses and contributed to the manuscript draft.

HM contributed to the coordination of the work and the manuscript draft.

FB initiated the work, contributed to sequence analyses, and wrote the last version of the manuscript.

All authors read and approved the final manuscript.

## Supplementary Material

Additional file 1 **Global alignment of *PPR-1*, *PPR-2*, *PPR-A*, *PPR-B*, and *PPR-C *gene sequences**. The alignment was performed on the Phylogeny.fr platform and edited with JALVIEW [69]. The residues are coloured according to percentage identity, from dark blue: 100% identity to white: less than 50% identity. The intron sequences determined from sequencing of RT-PCR products are shown in lower case. Click here for file

Additional file 2 **Maximum likelihood phylogenetic tree resulting from a multiple alignment of PPR repeat coding sequences of the 5 radish *Rfo-PPR *genes and the rapeseed *PPR-B-LIKE1 *gene**. Legend is as for Figure 8. Click here for file
