# Supplementary material for: Sequence analysis of two alleles reveals that intra-and intergenic recombination played a role in the evolution of the radish fertility restorer (Rfo)
Source: BMC Plant Biol. 2010 Feb 24;10:35. doi: 10.1186/1471-2229-10-35 (PMC2848758; doi:10.1186/1471-2229-10-35)
Supplement: Additional file 1 — Global alignment of PPR-1, PPR-2, PPR-A, PPR-B, and PPR-C gene sequences. The alignment was performed on the Phylogeny.fr platform and edited with JALVIEW [69]. The residues are coloured according to percentage identity, from dark blue: 100% identity to white: less than 50% identity. The intron sequences determined from sequencing of RT-PCR products are shown in lower case. [file 1471-2229-10-35-S1.PDF]

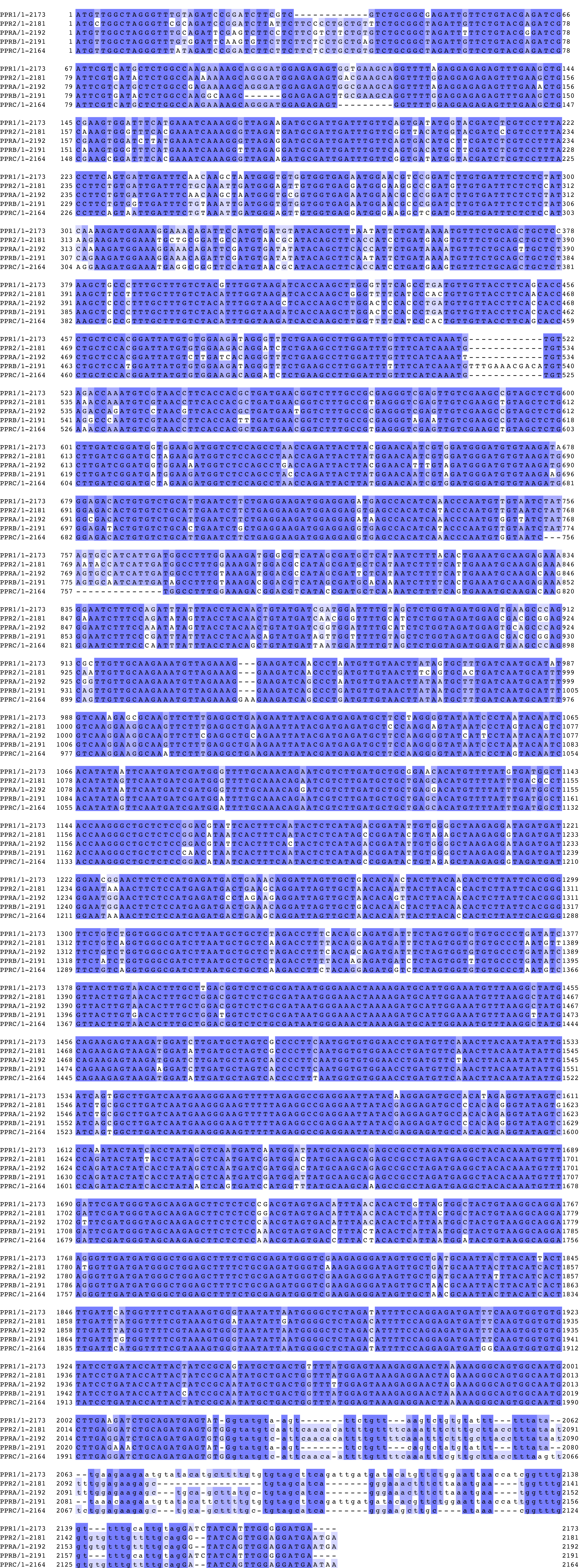
PPR1/1-2173
PPR2/1-2181
PPRA/1-2192
PPRB/1-2191
PPRC/1-2164
