# Supplementary figures and images for: Sequence analysis of two alleles reveals that intra-and intergenic recombination played a role in the evolution of the radish fertility restorer (Rfo)
Source: BMC Plant Biol. 2010 Feb 24;10:35. doi: 10.1186/1471-2229-10-35 (PMC2848758; doi:10.1186/1471-2229-10-35)

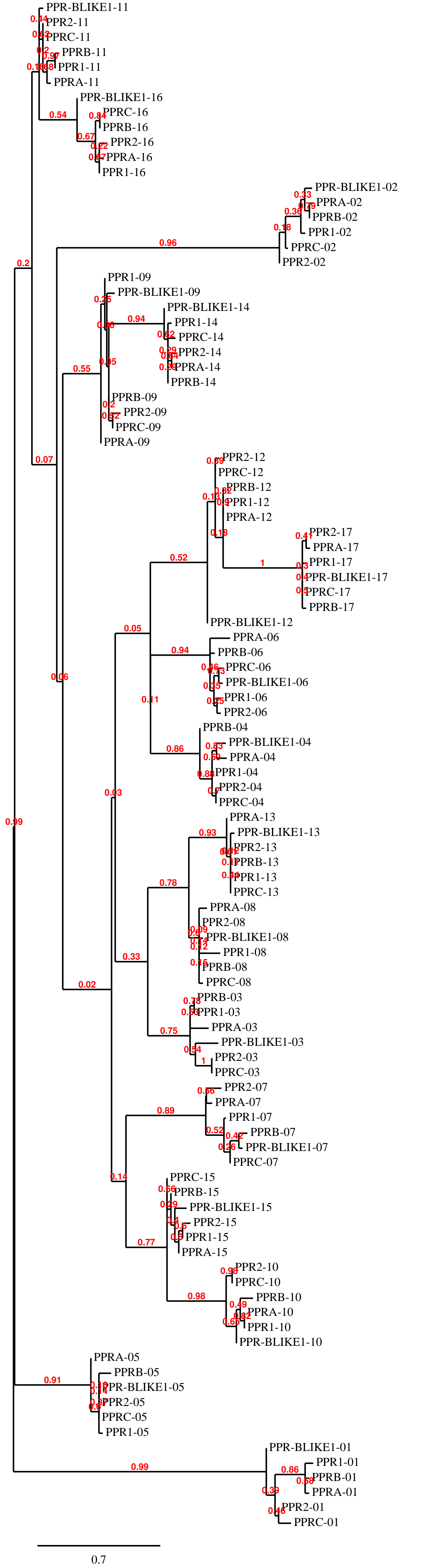

Supplement: Additional file 2 — Maximum likelihood phylogenetic tree resulting from a multiple alignment of PPR repeat coding sequences of the 5 radish Rfo-PPR genes and the rapeseed PPR-B-LIKE1 gene. Legend is as for Figure 8. [file 1471-2229-10-35-S2.PDF]
